# Whole Wheat Crackers Fortified with Mixed Shrimp Oil and Tea Seed Oil Microcapsules Prepared from Mung Bean Protein Isolate and Sodium Alginate

**DOI:** 10.3390/foods11020202

**Published:** 2022-01-12

**Authors:** Saqib Gulzar, Krisana Nilsuwan, Navaneethan Raju, Soottawat Benjakul

**Affiliations:** International Center of Excellence in Seafood Science and Innovation, Faculty of Agro-Industry, Prince of Songkla University, Songkhla 90110, Thailand; sgwani@hotmail.com (S.G.); krisana.n@psu.ac.th (K.N.); alexnaveen3@gmail.com (N.R.)

**Keywords:** shrimp oil, tea seed oil, mung bean protein, encapsulation, oxidation, cracker

## Abstract

Shrimp oil (SO) rich in n-3 fatty acids and astaxanthin, mixed with antioxidant-rich tea seed oil (TSO), was microencapsulated using mung bean protein isolate and sodium alginate and fortified into whole wheat crackers. SO and TSO mixed in equal proportions were emulsified in a solution containing mung bean protein isolate (MBPI) and sodium alginate (SA) at varied ratios. The emulsions were spray-dried to entrap SO-TSO in MBPI-SA microcapsules. MBPI-SA microcapsules loaded with SO-TSO showed low to moderately high encapsulation efficiencies (EE) of 32.26–72.09% and had a fair flowability index. Two selected microcapsules with high EE possessed the particle sizes of 1.592 and 1.796 µm with moderate PDI of 0.372 and 0.403, respectively. Zeta potential values were −54.81 mV and −53.41 mV. Scanning electron microscopic (SEM) images indicated that microcapsules were spherical in shape with some shrinkage on the surface and aggregation took place to some extent. Fourier transform infrared (FTIR) and differential scanning calorimetry (DSC) analyses of samples empirically validated the presence of SO-TSO in the microcapsules. Encapsulated SO-TSO showed superior oxidative stability and retention of polyunsaturated fatty acids (PUFAs) to unencapsulated counterparts during storage of 6 weeks. When SO-TSO microcapsules were fortified in whole wheat crackers at varying levels (0–10%), the crackers showed sensorial acceptability with no perceivable fishy odor. Thus, microencapsulation of SO-TSO using MBPI-SA as wall materials could be used as an alternative carrier system, in which microcapsules loaded with PUFAs could be fortified in a wide range of foods.

## 1. Introduction

Food lipids have been engineered to enhance their stability towards lipid oxidation. Microencapsulation is a potential technique, which has been successfully applied to protect the sensitive core material from the environment. Marine oils, susceptible to oxidation such as shrimp oil and fish oil, have been encapsulated to enhance their storage stability [1,2]. Shrimp oil has been previously encapsulated using the spray-drying method, in which carboxymethyl cellulose, lecithin, and fumed silica were used as wall materials [3].

Shrimp oil, extracted from the inedible portion of shrimp, is a rich source of astaxanthin, which is reported to possess 100 times higher antioxidant property than α-tocopherol [4]. Shrimp oil also contains high amounts of polyunsaturated fatty acids (PUFAs), particularly eicosapentaenoic acid (EPA) and docosahexaenoic acid (DHA) [5]. PUFAs are known to improve health conditions related to CVD and Alzheimer disease [6]. Tea seed oil has been traditionally used as medicine for several diseases including stomachache and burn injuries as well as antitussive and anthelmintic in China [7]. Due to its high polyphenol and flavonoid content, tea seed oil has superior anti-inflammatory, anticancer, and antithrombotic effects [8,9]. It is also known for preventing obesity and lowering cholesterol [10]. In a recent study, tea seed oil was reported to contain diverse phenolic compounds having a strong antioxidant capacity [11].

Food fortification is a cost-effective intervention to address the issues of nutrition deficiency globally as well as the value addition of foods to target a specific population [12]. Functional foods containing omega-3 fatty acids are one of the fastest-growing food product categories in the United States and Europe. Numerous products fortified with omega-3 fatty acids are present in markets all over the world [13]. Owing to the remarkable health benefits of shrimp oil and tea seed oil, they can be fortified in many food systems as nutraceuticals. However, shrimp oil has a large amount of PUFAs prone to oxidation along with a fishy odor. The blending of shrimp oil with tea seed oil rich in antioxidative compounds could lower oxidation. Blended sunflower oil and *Moringa oleifera* oil were documented to enhance oxidative stability of resulting oils [14]. In addition, a carrier system is necessary to protect the oils from oxidative deterioration and simultaneously to mask the fishy odor. Spray-drying microencapsulation has been successfully used as a delivery system for the entrapment of sensitive compounds. The spray-drying process is economical; however, it can lead to substantial variation in the microencapsulation matrix as well as the size and surface of microcapsules [15]. Spray drying of shrimp oil nanoliposomes resulted in the formation of stable microcapsules with high solubility and flowability [3].

Proteins and carbohydrates have been used extensively as wall materials in spray-drying microencapsulation [16]. Proteins are amphiphilic in nature with the emulsifying property. They can form stable oil-in-water emulsions. Moreover, upon drying of these emulsions, the proteins form a dense network around the oil, thus encapsulating the core. A combination of proteins and polysaccharides was reported to yield higher encapsulation efficiency [17]. The use of plant-based proteins is being recommended all over the world because of their low cost, high abundance, low allergenicity, and wide acceptability [18]. Legume proteins have extensive uses in the functional food industry [19]. Mung bean protein has significant functional properties that can be used for several food processing applications [20]. Sodium alginate, as a hydrocolloid, has also been used in the food industry, particularly for making complexes via coacervation with proteins [21]. However, no information on the use of mung bean protein isolate and sodium alginate as wall materials for encapsulation of shrimp oil is available.

Whole wheat cracker is a popular food containing dietary fibers. It has gained increasing interest due to its crunchy texture and health benefits. With the addition of shrimp oil and tea seed oil, the nutritional quality of the crackers can be further enhanced and promoted by nutraceutical properties associated with the increased essential fatty acids. Therefore, this study aimed to encapsulate the mixed shrimp oil and tea seed oil using mung bean protein and sodium alginate as wall materials by spray-drying and to characterize the resultant microcapsules. Mixed shrimp oil/tea seed oil microcapsules were also fortified into whole wheat crackers and sensorially characterized.

## 2. Materials and Methods

### 2.1. Materials

Shrimp oil (SO) was extracted from Pacific white shrimp cephalothorax following the method of Gulzar and Benjakul [22]. Tea seed oil (TSO) from Camellia oleifera seeds was procured from a local market. Sodium alginate (SA) (seaweed origin) was purchased from Qingdao Mingyue Seaweed Group Co., Ltd., Qingdao, China.

### 2.2. Preparation of Mung Bean Protein Isolate

Mung bean protein isolate (MBPI) was prepared following the method of El Adawy [23]. Briefly, mung beans were milled to obtain a fine flour and subsequently sieved to remove the coarse particles. The mung bean flour was defatted by mixing with butyl alcohol at a flour/solvent ratio of 1:10 (*w*/*v*) and stirred for 1 h. Defatted mung bean flour was dispersed in distilled water (DW) at 5% (*w*/*v*). The pH was adjusted to 9 using 0.1 N NaOH and the mixture was shaken for 1 h at room temperature. The dispersion was centrifuged at 10,000 *g* for 15 min at 4 °C. The supernatant was collected, followed by pH adjustment to 4 using 2 M HCl and centrifugation at 10,000 *g* for 15 min at 4 °C, respectively. The precipitate was washed with DW and lyophilized.

### 2.3. Preparation of MBPI/SA/SO-TSO Emulsions

MBPI (1, 2, and 3 g) was dispersed in 100 mL of DW, and pH was adjusted to 10 using 0.1 M NaOH to solubilize MBPI. SA was added at the fixed MBPI/SA ratio of 10:1 (*w*/*w*). Finally, the mixed SO/TSO (1:1), so-called SO-TSO, was added to the above solutions at 0.5, 0.75, and 1 g under vigorous stirring at 30 °C for 1 h, in which a homogenous mixture was obtained.

### 2.4. Spray-Drying Microencapsulation of MBPI/SA/SO-TSO Emulsions

The MBPI/SA/SO-TSO emulsions were spray-dried using a laboratory-scale spray-dryer (LabPlant Ltd., LabPlant SD-05, Huddersfield, UK). The sample was fed to the drying chamber by a peristaltic pump at the feed rate of 5 mL min^−1^. The inlet temperature was kept at 180 ± 2 °C at the airflow rate of 4.3 m s^−1^ and the outlet temperature was 105 ± 2 °C. Spray-dried powders or microcapsules with varying wall materials (MBPI and SA) and SO-TSO ratios including MBPI:SA:SO-TSO ratios of 1:0.1:0.5, 1:0.1:0.75, 1:0.1:1, 2:0.2:0.5, 2:0.2:0.75, 2:0.2:1, 3:0.3:0.5, 3:0.3:0.75, and 3:0.3:1 (*w*/*w*/*w*) were labelled as MC1, MC2, MC3, MC4, MC5, MC6, MC7, MC8, and MC9, respectively.

### 2.5. Characterization of Microcapsules

#### 2.5.1. Encapsulation Efficiency

Encapsulation efficiency (EE) of microcapsules loaded with SO-TSO was measured following the method of Takeungwongtrakul et al. [24]. Surface oil and total oil from powders were recovered and quantified. Surface oil was recovered by mixing 2 g of powder with 15 mL of hexane. The mixture was shaken by a vortex mixer (G-560E, Vortex Genie 2, Scientific Industries, Inc., Bohemia, NY, USA) at room temperature for 2 min. Filtration was carried out through a Whatman No. 1 filter paper. The extraction of surface oil was carried out three times. The hexane was pooled and placed in a round-bottom flask and evaporation of the solvent was done. The total oil was measured by dissolving 2 g of powder in 25 mL of 0.88% (*w*/*v*) KCl solution + 25 mL of methanol and 50 mL chloroform. The mixtures were homogenized for 5 min at 15,000 rpm and transferred to a separating funnel. The chloroform layer was collected, and chloroform was removed by evaporation using a rotary evaporator. EE was then calculated as follows (1):

(1) $$EE\;\left(\%\right)=\frac{TO-SO}{TO}\times 100$$

where *TO* and *SO* are total oil content and surface oil content, respectively.

#### 2.5.2. Flowability

Flowability of microcapsules loaded with SO-TSO was determined using the Carr index (*Ci*) calculated by the following equation (2):

(2) $$Ci\;\left(\%\right)=\frac{\left(\rho t-\rho u\right)}{\rho t}\times 100$$

where ρt is the tapped density of the microcapsule samples and ρu is the untapped density of microcapsules. Tapped density is measured by filling a graduated cylinder with the microcapsules and mechanically tapping the cylinder up and down 50 times against the table. The volume of the microcapsules was calculated from the graduations of the cylinder when no further change in volume took place after tapping of the cylinder. Tapped density was then calculated. Untapped density is the normal bulk density of microcapsules, calculated as mass per volume of microcapsules without any tapping.

### 2.6. Characterization of SO-TSO Microcapsules

Microcapsules using the optimal ratio of MDPI, SA, and SO-TSO that yielded the desirable encapsulation efficiency and flowability were selected for characterization.

#### 2.6.1. Particle Size, Poly-Dispersity Index (PDI), and Zeta Potential

Particle size, poly-dispersity index (PDI), and zeta (ζ) potential of SO-TSO microcapsules were determined using a PALS Zeta potential analyzer (Brookhaven instruments corp, Holtsville, NY, USA). Microcapsule samples were suitably diluted in ethanol and measured at 25 °C for size, PDI, and ζ potential.

#### 2.6.2. Microstructure

Microstructures of microcapsules loaded with SO-TSO were analyzed using a scanning electron microscope (SEM) (Quanta 400, FEI, Eindhoven, the Netherlands). The samples were mounted on individual bronze stubs and sputter-coated with a gold layer (Sputter coater SPI-Module, West Chester, PA, USA). The samples were visualized at an acceleration voltage of 20 kV and 5–10 Pa pressure. Magnifications from 10,000 to 100,000× were used.

#### 2.6.3. FTIR Spectra

MBPI, SA, SO-TSO, and the selected microcapsules loaded with SO-TSO were analyzed for their functional groups using an FTIR spectrometer (Bruker Model Equinox 55, Bruker Co., Ettlingen, Germany). Spectra of the mid-infrared region (4000–400 cm^−1^) were collected in 32 scans at a resolution of 4 cm^−1^.

#### 2.6.4. Differential Scanning Calorimetry

Differential scanning calorimeter (Perkin Elmer, Model DSC7, Norwalk, CA, USA) was used. Accurately weighed samples were loaded onto aluminum pans and sealed. Temperature scanning was performed at 10 °C/min over the range of −40 to 250 °C.

### 2.7. Oxidative Stability of SO-TSO and Microcapsules Loaded with SO-TSO

SO-TSO samples and selected microcapsules placed in sealed polythene pouches were stored at room temperature for 6 weeks. A SO-TSO sample and oil from microcapsules were tested for lipid oxidation at weeks 0 and 6. Before analysis, the total oil from the microcapsules was extracted using the method described previously.

#### 2.7.1. Lipid Oxidation

Peroxide value (PV) and thiobarbituric acid reactive substances (TBARS) were measured by the titration method, as described by Pudtikajorn and Benjakul [25] and the method as tailored by Buege and Aust [26], respectively.

#### 2.7.2. Fatty Acid Profile

Fatty acid profile expressed as fatty acid methyl esters (FAMEs) was determined using gas chromatography (GC), as detailed by Gulzar and Benjakul [27]. Briefly, lipid samples (10 mg) were dissolved in 1 mL of hexane and esterified with 200 µL of 2 M methanolic sodium hydroxide at 50 °C for 5 min. The mixture was cooled and added with 200 µL of 2 M methanolic hydrochloric acid. The prepared mixture was vortexed thoroughly and then centrifuged at 3500× *g* for 10 min. The hexane phase was collected and injected into gas chromatography (Agilent GC 7890B; Santa Clara, CA, USA). Injection temperature was maintained at 250 °C and the initial column temperature was first reduced to 80 °C. The temperature was increased at 4 °C min^−1^ ramp for 40 min to 220 °C and finally reached 240 °C. The eluted compounds were detected by a flame ionisation detector (FID) at 270 °C as a detector temperature. The chromatographic peaks of the samples were identified from the retention times of FAME, compared to those of standards (Supelco 37 Component FAME Mix). Fatty acid content was calculated based on the peak area ratio and expressed as a percentage.

### 2.8. Fortification of Selected Microcapsules Loaded with SO-TSO in Whole Wheat Crackers

#### 2.8.1. Preparation of Whole Wheat Crackers Fortified with Microcapsules

The whole wheat crackers were prepared following the method of Benjakul and Karnjanapratum [28]. Selected microcapsules loaded with SO-TSO were blended with whole wheat flour at a concentration of 0, 2.5, 5, 7.5, and 10% (*w*/*w*). The ingredients were mixed in a dough mixer (KitchenAid casserole multifunctional 5 k, KitchenAid, Benton Harbor, MI, USA) for 3 min and thereafter added with water and mixed at low speed for 3 min. The dough was sheeted and cut into the desired shape with a thickness of 0.4 mm. Finally, the shaped cracker dough was baked in an electric oven (Mamaru MR-1214, Mamaru Co., Ltd., Bangkok, Thailand) at 180 °C for 15 min. Wheat crackers were cooled and packed in zipped polythene bags.

#### 2.8.2. Sensory Evaluation of Whole Wheat Crackers

Sensory analysis of whole wheat crackers fortified with SO-TSO was performed following the method of Meilgaard et al. [29]. Each sample was assigned a random three-digit code and served in white plastic trays at room temperature under the fluorescent daylight-type illumination. Sixty non-trained panelists, who were familiar with whole wheat cracker consumption (age 25–35 years), took part in sensory evaluation. The panelists were asked to evaluate for appearance, color, fishy odor, rancid flavor, texture, taste, and overall likeness of fortified whole wheat crackers using a 9-point hedonic scale (1, extremely dislike; 9, extremely like). Panelists were asked to rinse their mouths after evaluating each sample.

#### 2.8.3. Chemical Composition of Selected Whole Wheat Cracker

Crackers fortified with microcapsules with the highest sensory acceptability score and a control sample (without microcapsules) were analyzed for moisture, protein, fat, and ash contents, using analytical method Nos. of 925.45(A), 981.10, 948.15, and 923.03, respectively [30]. Oil from the selected cracker was extracted and subjected to fatty acid profile analysis, as explained above.

### 2.9. Statistical Analysis

A completely randomized design was used for this study. Experiment and analysis were done in triplicate. ANOVA was performed using the SPSS software (Statistical Package for Social Science, IBM software, New York, NY, USA). Duncan’s multiple range test and t-test were used for mean comparison.

## 3. Results and Discussion

### 3.1. Encapsulation Efficiency (EE) and Flowability

EE of microcapsules loaded with SO-TSO using MBPI and SA as wall materials ranged between 32.26 and 72.09% (Table 1). The highest EE was found in the sample using MBPI:SA:SO-TSO of 3:0.3:0.5, while the lowest EE was obtained in the sample using MBPI:SA:SO-TSO (1:0.1:1). It was implied that a higher protein-to-oil ratio was directly proportional to the higher EE of powder samples. EE is highly influenced by the solubility of the protein, which subsequently affects the emulsifying properties of the protein. The high solubility of protein results in the extensive diffusion of protein chains to the oil/water interface and consequently stabilizes the small droplets of oil [31]. During spray-drying, the droplets are transformed into aerosols and the oil is intensively distributed inside them. Upon the evaporation of water from the aerosols, the oil is effectively encapsulated inside the surrounding protein acting as a wall material [32]. Mung bean protein is reported to have a solubility of 70.6% at pH 10 [33]. Apart from the hydrophobic protein–protein interactions, which stabilized the wall, the addition of an anionic polysaccharide (sodium alginate) resulted in the formation of a protein-anionic polysaccharide complex by electrostatic interactions, thereby promoting cooperative adsorption of protein-polysaccharide at the interface of oil droplets, which could enhance the EE [34]. Nevertheless, anionic polysaccharides also acted as protective hydrocolloids by inhibiting the aggregation and precipitation of charged dispersed proteins, thus increasing the soluble protein-anionic polysaccharide complexes [34]. Incorporation of a polysaccharide into the emulsion also provides stability by thickening the aqueous phase surrounding the oil droplet and delaying the coalescence of oil globules [17]. The combination of protein and polysaccharide results in the formation of a multilayer emulsion in which protein forms the first layer due to its high surface activity, whereas the polysaccharide forms the subsequent layer, which provides strong steric and electrostatic repulsion [35]. In another study, multilayer emulsions were formed by whey protein isolate and sodium alginate for microencapsulation of flaxseed oil [36]. Multilayer emulsions can significantly prevent the degradation of encapsulated compounds. Chitosan-pectin multilayer emulsions showed 3–4 times less degradation of astaxanthin during storage than conventional emulsions [37]. Protein-polysaccharide complexes have been exploited for stabilizing polyunsaturated fatty acids [38] and microencapsulation of flavor oils in cheese [39]. The results indicated that the molecular interactions between protein-polysaccharides and their relative concentrations significantly influenced the encapsulation of SO-TSO.

Flowability of powders corresponds to the free-flowing nature of the powders and is considered an important functional property of food powders. Carr index is reliable for determining the flowability of powders. The lower C*i* values correspond to the higher flowability of powders [40]. High hygroscopicity and the presence of surface oil on the microcapsules led to the formation of agglomerates. Agglomeration of microcapsules was undesirable since it reduced the solubility and wettability of microcapsules upon rehydration. It was observed that the microcapsules with low EE had high C*i* (Table 1), indicating that the microcapsules contained more surface oil and were more likely to form the agglomerates. MC7 showed the highest C*i* value, while MC3, MC5, MC6, and MC9 had the lowest C*i* value (*p* < 0.05). It was noted that microcapsules with high MBPI content in the wall exhibited high flowability as ascertained by low C*i* values. Milk powders with high surface fat were more cohesive, compared to low-fat milk powders [41]. Overall, the microcapsules were free-flowing with little agglomeration, particularly in the samples with high EE.

Based on the results, the ratio of wall materials used had a significant effect on the EE and flowability of the microcapsules. The low EE of microcapsules with a lower oil/wall ratio could be attributed to the insufficient wall material to produce a sufficiently strong structural matrix, thinner layer of wall material between encapsulated oil, and/or destabilization of weak emulsion droplets during spray drying [42]. It was reported by Ramakrishnan et al. [43] that the EE values of microcapsules with fish oil/wall ratio of 1:1 was in the range of 30–40% and it was increased to more than 70% when the oil/wall ratio was increased to 1:3. Based on high EE and flowability of spray-dried SO-TSO microcapsules, the MC3 (MBPI:SA:SO-TSO = 3:0.3:0.5) and MC6 (MBPI:SA:SO-TSO = 3:0.3:0.75) samples were further characterized for their physical and chemical properties.

### 3.2. Particle Size, Poly-Dispersity Index (PDI), and Zeta Potential

Particle sizes of MC3 and MC6 samples are tabulated in Table 2. Both samples were micrometer in size. The MC3 sample was larger in size as compared to MC6. The variation in the size of samples was related to the difference in EE of the samples, which affected the amount of oil loaded per gram of wall material. The MC3 samples had higher EE (Table 1). Consequently, a higher amount of SO-TSO loaded per gram of MBPI-SA was attained, making the microcapsules larger in size. Gulzar et al. [44] documented that chitosan nanoparticles loaded with shrimp oil having high EE were larger in size than those with lower EE. MC3 and MC6 samples had PDI of 0.403 and 0.372, respectively (Table 2). PDI is indicative of heterogeneity or the size distribution of particles in a system. Values below 0.05 are considered as monodisperse and the PDI values above 0.7 indicate that the samples have a broad size distribution [45]. Based on PDI, the samples were moderately distributed in size. Variation in particle size could occur due to several reasons including an uneven drying rate, non-uniform atomization associated with varying droplet size, emulsion stability, and wall material composition [46,47,48]. In the case of spray-drying microencapsulation, high PDI is not uncommon, due to the aforementioned reasons. Agustinisari et al. [49] reported the PDI of 0.468–0.705 for spray-dried whey protein-maltodextrin conjugates and chitosan microcapsules loaded with eugenol. In another study, PDI values of 0.288–0.530 were obtained in the spray-drying microencapsulation of sardine oil using vanillic acid-grafted chitosan as wall material [50]

The ζ potential values of MC3 and MC6 are shown in Table 2. The MC3 sample presented a slightly higher ζ potential value than the MC6 sample. This could be attributed to the smaller size of MC6, in which positively charged residues of proteins could be neutralized by alginate (anionic) polymers to a higher extent. Ofir et al. [51] reported that the absolute values of ζ potential were affected by the particle size of colloidal suspension. Gulzar and Benjakul [3] found higher negative ζ potential values of freeze-dried shrimp oil nanoliposomes with larger particle sizes, compared to the spray-dried counterparts. MC3 and MC6 samples showed negative values of ζ potential, indicating the presence of a large amount of negatively charged moieties on the surface of the powders. At neutral pH used for emulsion preparation, proteins become mostly negatively charged since the pH is higher than pI. Mung bean protein was reported to have a pI of 5 [52]. Overall, higher values of ζ potential signify higher stability of the microcapsules due to increased electrostatic repulsions among the particles, leading to less agglomeration or flocculation of the powders [3].

### 3.3. Microstructure

**Figure 1:**
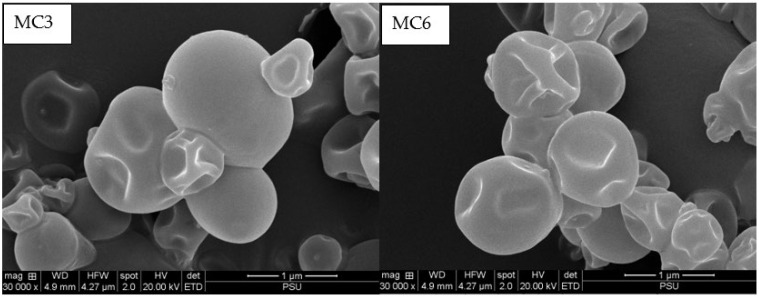
Scanning electron micrographs of spray-dried microcapsules loaded with SO-TSO having MBPI and SA as wall materials. SO-TSO: mixture of shrimp oil-tea seed oil (1:1); MBPI: mung bean protein isolate; SA: sodium alginate; MC3: spray-dried microcapsules containing 3% MBPI, 0.3% SA, and 0.5% (*w*/*w*) SO-TSO; MC6: spray-dried microcapsules containing 3% MBPI, 0.3% SA, and 0.75% (*w*/*w*) SO-TSO.

SEM micrographs of MC3 and MC6 samples are illustrated in Figure 1. Both samples were spherical with uneven surface indentation. Surface dents or wrinkles are generally caused by uneven drying rates, the nature and composition of wall materials, and atomization [53]. Generally, a rapid drying rate at high temperature affects the surface morphology of spray-dried powders, mainly caused by the differential shrinkage of the surface and core [54,55]. Spray drying is a complicated process in which any single droplet may experience a unique temperature-humidity environment as the drying progresses. It was reported by Andersson et al. [56] that the surface shrivels (wrinkles) in spray-dried microcapsules prepared from the native milk serum proteins were caused by the differential diffusion rates of protein aggregates found underneath the outer protein layer. It was also observed that MC3 and MC6 samples underwent aggregation to some degree. This could plausibly be caused by the high outlet drying temperature, which is above the wall material’s glass transition (Tg) [57]. Sodium alginate polymer, with a Tg at 81 °C, was reported to form strong inter- and intramolecular hydrogen bonds in the film when blended with starch [58]. Aggregates could also be formed by the surface oil that adhered the microcapsules together. Similar observations for agglomerated spray-dried capsules incorporated with fish oil were reported by Binsi et al. [59]. Despite the formation of some aggregates, the flowability of the microcapsules was still acceptable as measured by the Carr index (Table 1).

### 3.4. FTIR Spectra

**Figure 2:**
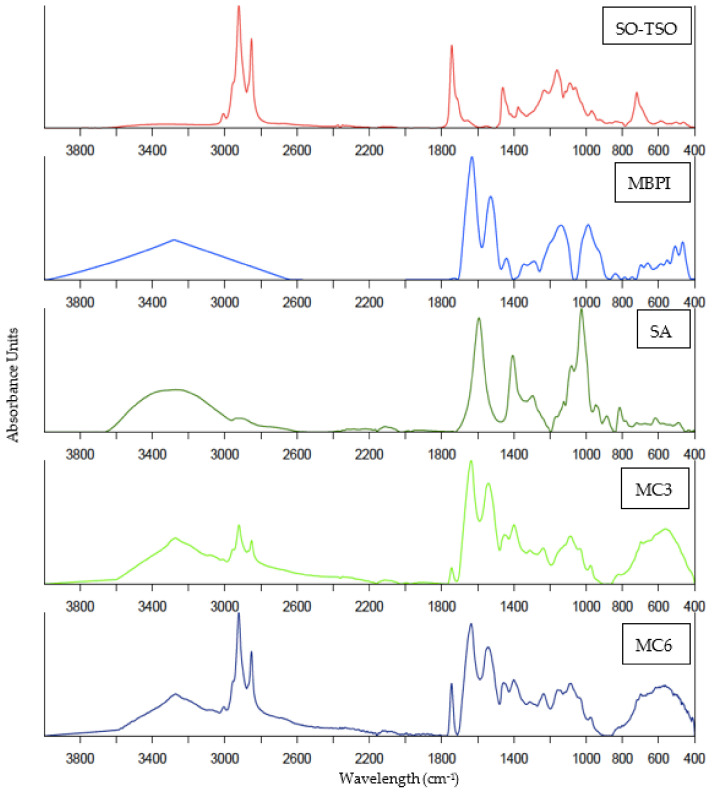
FTIR spectra of SO-TSO, MBPI, SA, MC3, and MC6 (for Captions, see Figure 1).

FTIR spectra of SO-TSO, MBPI, SA, MC3, and MC6 samples are illustrated in Figure 2. Characteristic stretching peaks in SO-TSO were found at 2920 cm^−1^ assigned to the –CH_2_– groups, which corresponded to the saturated fatty acid chains, and at 2850 cm^−1,^ corresponding to the aliphatic –CH– group representing the degree of unsaturation of oil [60]. Stretching vibrations were detected at 1750–1700 cm^−1^, corresponding to the esterified bonds between fatty acid chains and glycerol backbone in the SO-TSO sample [60]. Furthermore, the vibrations at ~1450 cm^−1^ could be correlated with the free fatty acids (FFA) present in the SO-TSO [61]. Depending upon the oxidation status and method of extraction of shrimp oil, FFA could be present between 9.11–34.9% in shrimp oil [5,22]. Asymmetric vibrations between ~1260–1150 cm^−1^ in the SO-TSO sample corresponded to the phosphate group of the phospholipid moiety. As reported by Gulzar and Benjakul [61], phospholipids were abundantly present in shrimp oil (45.98%). Several major bands were observed in the MBPI sample. Those peaks were characteristic of all protein isolates. Kudre et al. [33] reported that the peaks between 3411–3305 cm^−1^ of amide A of MBPI represented the free and hydrogen-bonded NH group of the protein. The peak at 1640 cm^−1^ corresponded to the amide I band of the secondary structure of legume proteins [62]. The peak at 1550 cm^−1^ associated with the amide II band of the MBPI represented the –NH bending and –CN stretching vibrations [63]. In addition, prominent peaks were also observed at 1160 cm^−1^ and 990 cm^−1^, which were attributed to the C–O stretching modes from ester bonds [64]. Large absorption bands in the range of 3600–3000 cm^−1^ were seen in the SA due to the stretching vibration band of –OH groups in the alginic acid. Observed bands at ~1600 cm^−1^ and 1400 cm^−1^ were attributed to the symmetric and symmetric stretching vibrations of the COO– groups, respectively [65]. Stretching C–C peak at ~1030 cm^−1^ suggested strong O–H binding vibration, which was characteristic of the guluronic acid present in the polymer chain of alginates [65]. In the case of MC3 and MC6 samples, characteristic peaks from SO-TSO, SA, and MBPI were observed in the spectra, indicating the presence of SO-TSO and MBPI in the microcapsule samples. Moreover, the peaks at 2920 cm^−1^, 2850 cm^−1^, and 1750 cm^−1^ were observed at a higher amplitude in MC6, implying the presence of a higher quantity of free SO-TSO especially on the surface in the sample, compared to MC3. Coincidentally, peaks that were characteristic of protein became lower in amplitude for MC6, indicating the lower ratio of proteins as the wall in this sample (Table 1). Therefore, the results obtained from FTIR spectra were handy in confirming the presence of SO-TSO in spray-dried microcapsules.

### 3.5. Differential Scanning Calorimetry

**Figure 3:**
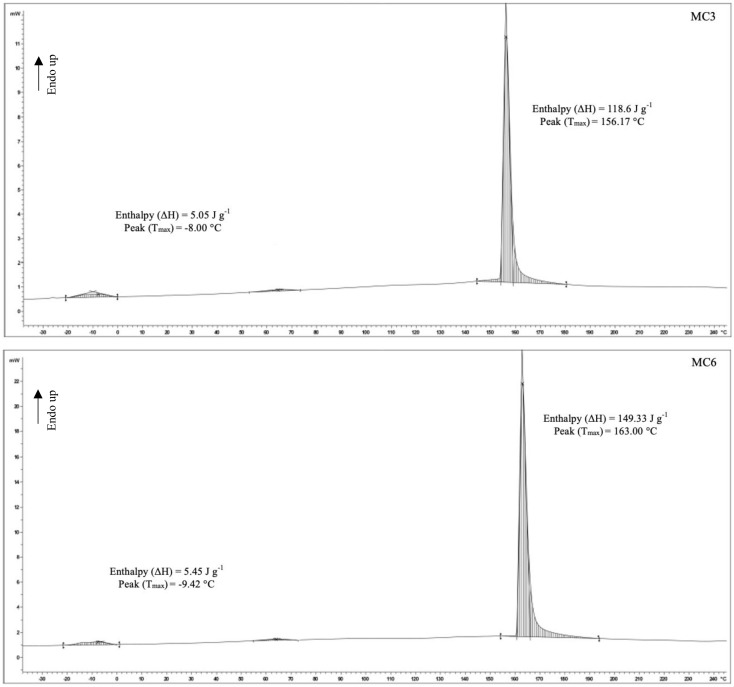
DSC thermograms of MC3 and MC6 (for Captions, see Figure 1).

DSC thermograms of MC3 and MC6 samples are illustrated in Figure 3. Sharp endothermic peaks were observed at 156.17 °C and 163 °C for MC3 and MC6 samples, respectively. These peaks depicted the denaturation of MBPI present in the microcapsule wall. In a study reported by Branch and Maria [66], DSC thermograms demonstrated denaturation temperature of MBPI at 157.9 °C. The higher endothermic peaks in the MC6 sample might be due to the presence of higher oil content in the sample (Table 1). Proteins in the wall or protein-alginate in the wall were stabilized by polar hydrogen bonds and non-polar hydrophobic interactions or ionic interactions [66]. Heat applied was able to destroy intermolecular bonds. Larger interactions among the molecules required more energy at higher temperatures to disrupt the bonds. In MC6 samples, the presence of more hydrophobic interactions between the SO-TSO and MBPI was postulated, as shown by higher T_max_. The difference in the enthalpy of denaturation of the two samples was also dependent on the temperature of denaturation. Differences in the protein denaturation temperatures were controlled majorly by nonpolar hydrophobic interactions and a type of “cooperativity” between the polar and nonpolar groups [67]. Smaller peaks in MC3 and MC6 at −8 °C and −9.42 °C, respectively, could be attributed to the transition of SO-TSO from crystalline to amorphous state (melting). Camellia oil was reported to have T_max_ at −6.65 °C, owing to the melting of oil [68]. Thus, microcapsules loaded with SO-TSO in MBPI-SA as wall material had varying thermal properties, depending on the ratio of mixed oil used in the formulation. Additionally, the interaction of oil and proteins in the wall or state of oil in the microcapsules affected the thermal stability of the resulting microcapsules.

### 3.6. Oxidative Stability of Microcapsules Loaded with SO-TSO

#### 3.6.1. Lipid Oxidation

**Figure 4:**
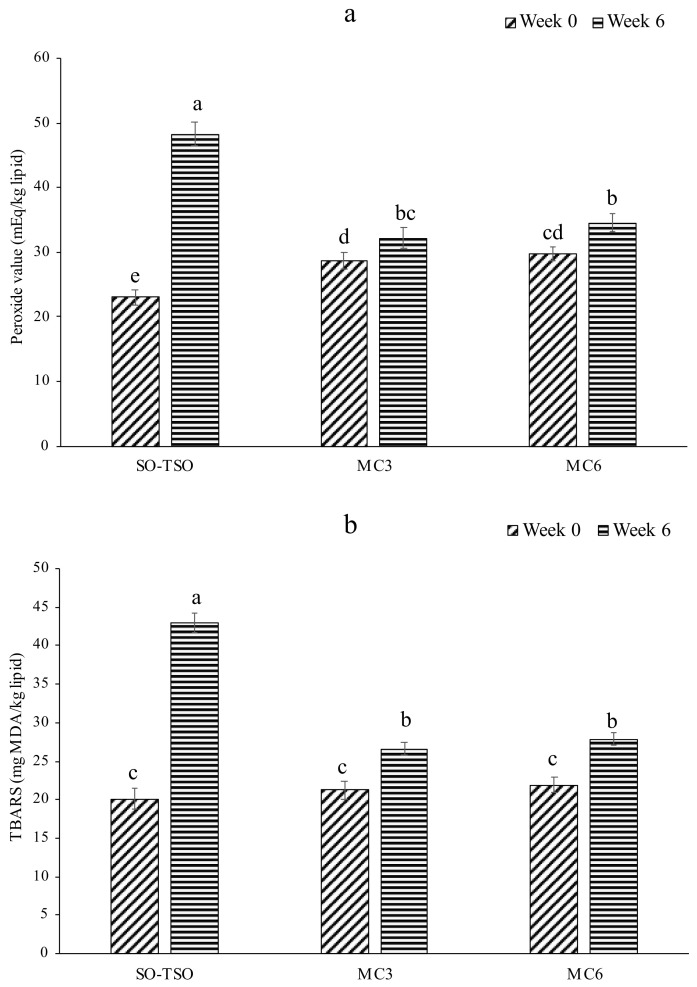
Peroxide value (**a**) and TBARS values (**b**) of SO-TSO, oil extracted from MC3, and oil extracted from MC6 stored for 0 and 6 weeks. Bars represent the standard deviation (*n* = 3). Different lowercase letters on the bars denote significant difference (*p* < 0.05) (for Captions, see Figure 1).

Peroxide value (PV) and TBARS value of SO-TSO and oil extracted from MC3 and MC6 before and after storage at room temperature for 6 weeks are depicted in Figure 4a,b. PV of oil extracted from MC3 and MC6 samples at week 0 was higher (*p* < 0.05) compared to that of SO-TSO, plausibly due to the exposure of oil to high temperature during spray-drying. However, oil extracted from both microcapsules showed lower PV than SO-TSO after 6 weeks of storage. The result suggested that encapsulation of SO-TSO in MBPI-SA microcapsules was effective in conquering the oxidative deterioration of the oil. Nevertheless, there was some increase in PV of oil from MC3 and MC6 samples after 6 weeks. This could be ascribed to the oxidation of surface oil present on microcapsules. Encapsulation of oil has been reported to retard oxidative deterioration by forming the barrier against oxygen and prooxidants [1,3,69,70]. Shrimp oil, in particular, is highly susceptible to oxidation due to the presence of oxygen-sensitive PUFAs [61]. TBARS values had a similar trend to that of PV. TBARS value of SO-TSO was augmented by 2 times over the storage period of 6 weeks, reflecting the quality deterioration of oil by the formation of secondary oxidation products. TBARS value of oil from MC3 and MC6 significantly rose (*p* < 0.05) after 6 weeks, most likely owing to the oxidation of surface oil of microcapsules. A higher TBARS value was found in MC6 than in MC3. This was related well with the higher surface oil of the former (lower EE). In addition to the protective effect of the wall, the antioxidants present in the SO-TSO also helped in the retardation of oxidation. Astaxanthin in SO reacts with the radicals, thus protecting the lipids from oxidation [71]. Phenolic compounds in TSO [11] also acted as an antioxidant to retard oxidation of PUFAs in SO. Overall, the quality of SO-TSO was well preserved from oxidation by the encapsulation using MBPI-SA as wall materials.

#### 3.6.2. Fatty acid Profile

Fatty acid profiles of SO-TSO and oils extracted from MC3 and MC6 before and after storage of 6 weeks are shown in Table 3. Oleic acid (C18:1) was found to be the predominant fatty acid in the SO-TSO. Oleic acid is the most abundant monounsaturated fatty acid (MUFA) present in TSO (56.98%) and SO (14.90%) [72,73]. Palmitic acid was the dominant saturated fatty acid (SFA). PUFAs in the SO-TSO were majorly contributed by the SO, which was rich in PUFAs, particularly DHA, EPA, and linoleic acid [73]. There was a sharp decline in the PUFA content of SO-TSO after 6 weeks of storage. The loss of PUFA content was caused by their rapid oxidation [61]. Compared to 36.67% loss in the PUFA content of SO-TSO after storage, the losses in the PUFA content of oil from MC3 and MC6 were 8.14% and 9.58%, respectively. The results demonstrated the high potency of microcapsules to retard the oxidation in the oils. Moreover, the antioxidants in SO-TSO inherently provided protection to the oil from oxidative deterioration. Nevertheless, additional protection such as the packaging of microcapsules was still necessary to maintain the quality of SO-TSO.

### 3.7. Acceptability of Whole Wheat Crackers Fortified with MC3

**Figure 5:**
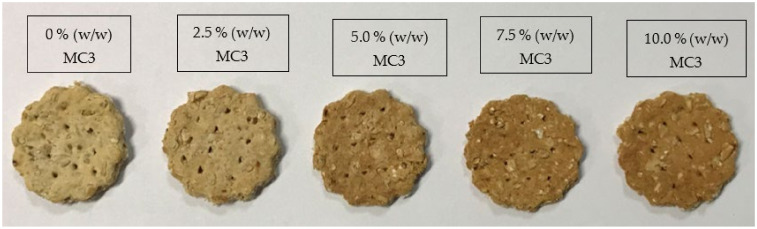
Whole wheat crackers fortified with MC3 at different levels. MC3: spray-dried microcapsules containing 3% MBPI, 0.3% SA, and 0.5% (*w*/*w*) SO-TSO.

Likeness scores of whole wheat crackers fortified with MC3 at 0, 2.5, 5, 7.5, and 10% (*w*/*w*) are tabulated in Table 4. There was no difference in the appearance and color likeness scores of the samples, irrespective of the amount of MC3 added (*p* > 0.05). For appearance, all samples were round in shape (Figure 5) with similar diameters. With the increase in MC3 levels, the crackers appeared more orange in color, due to the presence of an increased amount of SO, which contained the reddish-orange pigment astaxanthin (Figure 5). There was no perceivable fishy odor in all fortified samples, confirming that the inherent fishy odor in shrimp oil was completely masked by encapsulation. The rancid flavor was also within acceptable limits, as indicated by the high score. One of the most paramount advantages of encapsulating shrimp oil is the masking of an offensive fishy odor, which is highly disliked and makes fortification of shrimp oil difficult in foods [74]. Encapsulation of shrimp oil in nanoliposomes could significantly mask its fishy odor [75]. The texture of the crackers was neither liked nor disliked by the panelists. Incorporation of microcapsules up to 7.5% did not have a significant effect on the likeness score for texture (*p* > 0.05). However, the cracker fortified with 10% MC3 was found to be more crispy, which could be caused by the disruption of the gluten network by the microcapsules, especially when added at a higher level. In general, the texture scores were still above the acceptable level, in which a score of >5 was obtained. Likeness score for taste indicated that the taste of crackers was liked by the panelists. However, the sample fortified with 10% MC3 received the lowest score (*p* > 0.05). For overall likeness, all the samples except that fortified with 10% MC3 showed similar scores (*p* > 0.05). The sensory acceptability of MC3 fortified crackers became lowered when MC3 at 10% was incorporated (*p* < 0.05). Overall, encapsulation could be a promising carrier, which masked the fishy odor of SO-TSO effectively.

### 3.8. Chemical Composition of Cracker

Moisture, lipid, protein, and ash content of whole wheat crackers fortified with 0 and 7.5% MC3 are shown in Table 5. The moisture content of crackers containing microcapsules was lower than that of the control sample (*p* < 0.05). This could be attributed to the lower water retention in the dough as the amount of wheat was reduced and replaced by microcapsules. The results were concomitant with Benjakul and Karnjanapratum [28], who reported the decrease in the moisture content of whole wheat crackers when bio-calcium was added in a dose-dependent manner. The lipid and protein contents of crackers containing microcapsules were higher (*p* < 0.05), due to the presence of oil (SO-TSO) and MBPI in the microcapsules. Fat content was increased by 21.03%, while protein content was augmented by 27.65%. Ash content was higher in crackers fortified with 7.5% MC3, while carbohydrate was lower than those found in the control.

The fatty acid profile of the crackers fortified with 7.5% MC3 (Table 6) revealed the decline in EPA and DHA contents by 23.47 and 29.08%, respectively, compared to the EPA and DHA contents of SO-TSO. The loss of EPA and DHA could be caused by the oxidation of oil, especially on the surface of microcapsules by the high baking temperature of the crackers. Oil extracted from the crackers contained a high amount of linolenic acid followed by oleic and palmitic acids. For crackers fortified with 7.5% MC3, linolenic acid was predominant, followed by oleic and palmitic acids. EPA and DHA of oil extracted from crackers fortified with 7.5% MC3 were found at 1.63 and 4.59%, respectively. These two n-3 fatty acids were mainly from SO in the microcapsules. Crude lipid from whole wheat was found to contain 57.9% linoleic acid present principally in the bran [76]. Overall, the encapsulation of SO-TSO in MBPI-SA microcapsules was able to protect the oxidation of polyunsaturated fatty acids to a large extent.

## 4. Conclusions

SO-TSO was encapsulated by the combination of MBPI-SA using spray-drying. Emulsions formed by different combinations of MBPI-SA and SO-TSO ratios produced microcapsules of varying encapsulation efficiency upon spray-drying. Spherical microcapsules with some shrinkage on the surface were obtained. Microcapsules had moderate flowability. Encapsulation of SO-TSO resulted in enhanced oxidative stability and retention of PUFAs over 6 weeks of storage. Fortification of SO-TSO loaded microcapsules up to 7.5% in whole wheat crackers showed acceptable sensory attributes (overall likeness above 6). Therefore, the use of low-cost, plant-based proteins, especially mung bean protein isolate together with the abundantly available sodium alginate seaweed hydrocolloid, was effective for encapsulating oxidation-sensitive shrimp oil/tea seed oil mixture having excellent health benefits.

## Figures and Tables

**Table 1:** Encapsulation efficiency (EE) and Carr index (C*i*) of SO-TSO encapsulated using MBPI and SA at varied levels.

Sample	Wall Materials (*w*/*w*/*w*)	EE (%)	Carr Index (C*i*) (%)
MC1	MBPI:SA:SO-TSO (1:0.1:0.5)	45.27 ± 1.09 ^d,^*	23.92 ± 1.04 ^c^
MC2	MBPI:SA:SO-TSO (2:0.2:0.5)	65.61 ± 2.23 ^b^	22.25 ± 1.28 ^c,d^
MC3	MBPI:SA:SO-TSO (3:0.3:0.5)	72.09 ± 2.97 ^a^	20.02 ± 1.17 ^d^
MC4	MBPI:SA:SO-TSO (1:0.1:0.75)	33.74 ± 1.64 ^e^	27.61 ± 1.34 ^b^
MC5	MBPI:SA:SO-TSO (2:0.2:0.75)	56.44 ± 1.98 ^c^	21.31 ± 1.15 ^d^
MC6	MBPI:SA:SO-TSO (3:0.3:0.75)	63.62 ± 2.64 ^b^	21.18 ± 1.09 ^d^
MC7	MBPI:SA:SO-TSO (1:0.1:1)	32.26 ± 1.33 ^e^	31.63 ± 1.19 ^a^
MC8	MBPI:SA:SO-TSO (2:0.2:1)	43.22 ± 1.87 ^d^	27.09 ± 1.67 ^b^
MC9	MBPI:SA:SO-TSO (3:0.3:1)	58.29 ± 2.72 ^c^	21.39 ± 1.34 ^d^

Note: SO-TSO: a mixture of shrimp oil-tea seed oil (1:1); MBPI: mung bean protein isolate; SA: sodium alginate. Values are presented as mean ± SD (*n* = 3). * Different lowercase superscripts in the same column indicate the significant difference (*p* < 0.05).

**Table 2:** Particle size, poly-dispersity index (PDI), and zeta potential of spray-dried microcapsules loaded with SO-TSO having MBPI and SA as wall materials.

Sample	Size (µm)	PDI	Zeta Potential (mV)
MC3	1.796 ± 0.043 ^a^	0.403 ± 0.01 ^a^	−54.81 ± 0.53 ^a^
MC6	1.592 ± 0.043 ^b^	0.372 ± 0.01 ^b^	−53.41 ± 0.69 ^b^

Note: SO-TSO: mixture of shrimp oil-tea seed oil (1:1); MBPI: mung bean protein isolate; SA: sodium alginate; MC3: spray-dried microcapsules containing 3% MBPI, 0.3% SA, and 0.5% (*w*/*w*) SO-TSO; MC6: spray-dried microcapsules containing 3% MBPI, 0.3% SA, and 0.75% (*w*/*w*) SO-TSO. Values are presented as mean ± SD (*n* = 3). Different lowercase superscripts in the same column indicate the significant difference (*p* < 0.05).

**Table 3:** Fatty acid profile of SO-TSO, oil extracted from MC3, and oil extracted from MC6 on the 0th and 6th week of storage.

Fatty Acids (%)	Week 0			Week 6		
	SO-TSO	MC3	MC6	SO-TSO	MC3	MC6
C14:0 (Myristic)	1.32 ± 0.08 ^a^	1.33 ± 0.07 ^a^	1.34 ± 0.04 ^a^	1.36 ± 0.05 ^a^	1.34 ± 0.04 ^a^	1.37 ± 0.08 ^a^
C15:0 (Pentadecanoic)	0.47 ± 0.01 ^b^	0.49 ± 0.01 ^a,b^	0.50 ± 0.01 ^a^	0.51 ± 0.01 ^a^	0.50 ± 0.01 ^a^	0.51 ± 0.02 ^a^
C16:0 (Palmitic)	13.99 ± 0.27 ^b^	14.07 ± 0.24 ^b^	14.01 ± 0.22 ^b^	14.79 ± 0.18 ^a^	14.41 ± 0.21 ^a,b^	14.52 ± 0.19 ^a^
C16:1 (Palmitoleic)	1.61 ± 0.09 ^a^	1.59 ± 0.11 ^a^	1.57 ± 0.07 ^a^	1.27 ± 0.06 ^b^	1.51 ± 0.07 ^a^	1.50 ± 0.08 ^a^
C17:0 (Heptadecanoic)	1.09 ± 0.04 ^b^	1.11 ± 0.02 ^b^	1.13 ± 0.04 ^b^	1.21 ± 0.07 ^a^	1.14 ± 0.03 ^a,b^	1.16 ± 0.01 ^a,b^
C17:1 cis 10 (cis-10-Heptadecanoic)	0.31 ± 0.01 ^a^	0.30 ± 0.01 ^a^	0.28 ± 0.02 ^a,b^	0.19 ± 0.03 ^c^	0.28 ± 0.01 ^a,b^	0.26 ± 0.03 ^b^
C18:0 (Stearic)	4.98 ± 0.09 ^b,c^	4.93 ± 0.07 ^c^	4.95 ± 0.10 ^b,c^	5.99 ± 0.11 ^a^	5.05 ± 0.09 ^b,c^	5.11 ± 0.08 ^b^
C18:1 (Oleic)	50.55 ± 0.67 ^a^	50.24 ± 0.71 ^a^	50.36 ± 0.64 ^a^	48.27 ± 0.78 ^b^	50.02 ± 0.69 ^a^	49.82 ± 0.81 ^a^
C18:2 (Linoleic)	8.51 ± 0.19 ^a^	8.36 ± 0.25 ^a^	8.32 ± 0.22 ^a^	5.52 ± 0.21 ^c^	7.56 ± 0.19 ^b^	7.49 ± 0.18 ^b^
C18:3 (alpha-Linolenic)	0.91 ± 0.02 ^a^	0.89 ± 0.01 ^a^	0.88 ± 0.01 ^a^	0.41 ± 0.01 ^c^	0.78 ± 0.02 ^b^	0.77 ± 0.03 ^b^
C20:0 (Arachidic)	0.54 ± 0.01 ^c^	0.53 ± 0.01 ^c^	0.54 ± 0.01 ^c^	0.66 ± 0.01 ^a^	0.54 ± 0.01 ^c^	0.57 ± 0.01 ^b^
C20:1 (Eicosenoic)	0.82 ± 0.03 ^a^	0.81 ± 0.02 ^a^	0.78 ± 0.03 ^a^	0.71 ± 0.04 ^b^	0.79 ± 0.02 ^a^	0.77 ± 0.04 ^a^
C20:2 (Eicosadienoic)	0.56 ± 0.02 ^a^	0.54 ± 0.03 ^a,b^	0.53 ± 0.01 ^a,b,c^	0.36 ± 0.03 ^d^	0.50 ± 0.04 ^b,c^	0.49 ± 0.01 ^c^
C20:5 (EPA)	2.21 ± 0.12 ^a^	2.13 ± 0.11 ^a^	2.15 ± 0.09 ^a^	1.29 ± 0.14 ^b^	2.01 ± 0.09 ^a^	1.98 ± 0.15 ^a^
C22:6 (DHA)	6.58 ± 0.11 ^a^	6.47 ± 0.09 ^a^	6.45 ± 0.12 ^a^	4.40 ± 0.13 ^c^	6.04 ± 0.12 ^b^	5.91 ± 0.17 ^b^
C23:0 (Tricosanoic)	1.02 ± 0.07 ^a^	1.01 ± 0.05 ^a^	1.03 ± 0.01 ^a^	1.07 ± 0.04 ^a^	1.04 ± 0.06 ^a^	1.06 ± 0.01 ^a^
C24:1 (Nervonic)	0.31 ± 0.01 ^a^	0.30 ± 0.01 ^a,b^	0.30 ± 0.01 ^a,b^	0.26 ± 0.02 ^c^	0.29 ± 0.01 ^a,b^	0.28 ± 0.02 ^b,c^
Others	4.19 ± 0.12 ^b^	4.37 ± 0.20 ^a,b^	4.26 ± 0.18 ^b^	4.67 ± 0.24 ^a^	4.44 ± 0.17 ^a,b^	4.34 ± 0.16 ^a,b^
Saturated fatty acid (SFA)	23.71 ± 0.28 ^c,d^	22.97 ± 0.22 ^e^	23.49 ± 0.33 ^d^	25.62 ± 0.29 ^a^	24.01 ± 0.27 ^b,c^	24.31 ± 0.19 ^b^
Monounsaturated fatty acid (MUFA)	53.71 ± 0.72 ^a^	53.25 ± 0.63 ^a^	53.31 ± 0.52 ^a^	50.69 ± 0.66 ^b^	52.91 ± 0.54 ^a^	52.62 ± 0.67 ^a^
Polyunsaturated fatty acid (PUFA)	18.90 ± 0.29 ^a^	18.41 ± 0.27 ^b^	18.36 ± 0.22 ^b^	11.97 ± 0.19 ^d^	16.91 ± 0.25 ^c^	16.65 ± 0.23 ^c^

Note: SO-TSO: mixture of shrimp oil-tea seed oil (1:1); MBPI: mung bean protein isolate; SA: sodium alginate; MC3: spray-dried microcapsules containing 3% MBPI, 0.3% SA, and 0.5% (*w*/*w*) SO-TSO; MC6: spray-dried microcapsules containing 3% MBPI, 0.3% SA, and 0.75% (*w*/*w*) SO-TSO. Different lowercase superscripts in the same row indicate the significant difference (*p* < 0.05).

**Table 4:** Likeness scores of whole wheat crackers fortified with MC3 at different levels.

MC3 Level (%, *w*/*w*)	Appearance	Color	Fishy Odor **	Rancid Flavor **	Texture	Taste	Overall Likeness
0	7.20 ± 0.75 ^a^	6.80 ± 1.47 ^a^	7.80 ± 0.60 ^a^	8.10 ± 0.54 ^a^	5.90 ± 1.22 ^a,b^	6.30 ± 0.90 ^a^	6.40 ± 1.02 ^a,b^
2.5	7.10 ± 1.14 ^a^	6.80 ± 1.33 ^a^	7.70 ± 0.78 ^a^	7.70 ± 0.64 ^a^	5.90 ± 0.83 ^a,b^	6.00 ± 1.18 ^a^	6.50 ± 1.28 ^a,b^
5.0	6.80 ± 0.98 ^a^	6.50 ± 1.20 ^a^	7.40 ± 0.92 ^a^	7.60 ± 0.66 ^a^	6.20 ± 0.60 ^a,b^	6.40 ± 0.80 ^a^	6.40 ± 0.80 ^a,b^
7.5	6.80 ± 1.25 ^a^	6.50 ± 0.81 ^a^	7.80 ± 0.60 ^a^	7.50 ± 0.67 ^a^	6.40 ± 0.80 ^a^	6.10 ± 0.54 ^a^	6.60 ± 0.49 ^a^
10.0	6.80 ± 1.47 ^a^	6.40 ± 0.80 ^a^	7.50 ± 0.81 ^a^	7.50 ± 0.77 ^a^	5.50 ± 1.02 ^b^	5.60 ± 0.80 ^a^	5.70 ± 0.64 ^b^

Note: MC3: spray-dried microcapsules containing 3% MBPI, 0.3% SA, and 0.5% (*w*/*w*) SO-TSO; MBPI: mung bean protein isolate; SA: sodium alginate; SO-TSO: mixture of shrimp oil-tea seed oil (1:1). Different lowercase superscripts in the same column indicate the significant difference (*p* < 0.05). ** Higher score indicates lower fishy odor or rancid flavor.

**Table 5:** Chemical composition of whole wheat cracker and that fortified with 7.5% MC3.

MC3 Level (%, *w*/*w*)	Composition (%, *w*/*w*, Wet Basis)				
	Moisture **	Lipid	Protein (g/100 g)	Ash	Carbohydrate
0	3.05 ± 0.02 ^a^	2.90 ± 0.10 ^b^	12.84 ± 0.11 ^b^	3.23 ± 0.14 ^b^	77.97 ± 0.17 ^a^
7.5	2.06 ± 0.07 ^b^	3.51 ± 0.11 ^a^	16.39 ± 0.14 ^a^	4.28 ± 0.06 ^a^	73.77 ± 0.16 ^b^

Note: MC3: spray-dried microcapsules containing 3% MBPI, 0.3% SA, and 0.5% (*w*/*w*) SO-TSO; MBPI: mung bean protein isolate; SA: sodium alginate; SO-TSO: mixture of shrimp oil-tea seed oil (1:1). Different lowercase superscripts in the same column indicate the significant difference (*p* < 0.05). ** Wet weight basis.

**Table 6:** Fatty acid profiles of whole wheat cracker and that fortified with 7.5% MC3.

Fatty Acids (%)	MC3 Level (%, *w*/*w*)	
	0	7.5
C14:0 (Myristic)	ND	1.85 ± 0.09 ^a^
C15:0 (Pentadecanoic)	ND	0.89 ± 0.07 ^a,b^
C16:0 (Palmitic)	16.09 ± 0.21 ^b^	18.37 ± 0.44 ^a^
C16:1 (Palmitoleic)	1.37 ± 0.19 ^a^	1.56 ± 0.10 ^a^
C17:0 (Heptadecanoic)	ND	1.52 ± 0.13 ^a^
C18:0 (Stearic)	5.68 ± 0.13 ^a^	5.37 ± 0.08 ^b^
C18:1 (Oleic)	17.37 ± 0.42 ^b^	23.18 ± 0.52 ^a^
C18:2 (Linoleic)	4.96 ± 0.28 ^a^	6.42 ± 0.51 ^a^
C18:3 (alpha-Linolenic)	48.91 ± 0.49 ^a^	28.17 ± 0.17 ^b^
C20:0 (Arachidic)	0.67 ± 0.04 ^a^	0.33 ± 0.01 ^b^
C20:2 (Eicosadienoic)	ND	0.34 ± 0.01 ^a^
C20:5 (EPA)	ND	1.63 ± 0.17 ^a^
C22:6 (DHA)	ND	4.59 ± 0.19 ^a^
C23:0 (Tricosanoic)	ND	1.92 ± 0.08 ^a^
C24:1 (Nervonic)	ND	0.21 ± 0.01 ^a^
Saturated fatty acid (SFA)	22.46 ± 0.58 ^b^	26.15 ± 0.28 ^a^
Monounsaturated fatty acid (MUFA)	18.71 ± 0.42 ^b^	25.27 ± 0.63 ^a^
Polyunsaturated fatty acid (PUFA)	53.78 ± 0.36 ^a^	41.31 ± 0.31 ^b^

Note: SO-TSO: mixture of shrimp oil-tea seed oil (1:1); MBPI: mung bean protein isolate; SA: sodium alginate; MC3: spray-dried microcapsules containing 3% MBPI, 0.3% SA, and 0.5% (*w*/*w*) SO-TSO; ND: Not detected. Different lowercase superscripts in the same row indicate the significant difference (*p* < 0.05).

## Data Availability

Data are contained within the article.
